# Guamara and Cocuixtle: Source of Proteases for the Transformation of Shrimp By-Products into Hydrolysates with Potential Application

**DOI:** 10.3390/biology12050753

**Published:** 2023-05-21

**Authors:** Juan Miguel de Jesús Rodríguez-Jiménez, Efigenia Montalvo-González, Ulises Miguel López-García, Julio César Barros-Castillo, Juan Arturo Ragazzo-Sánchez, María de Lourdes García-Magaña

**Affiliations:** Laboratorio Integral de Investigación en Alimentos, Tecnológico Nacional de México/Instituto Tecnológico de Tepic, Avenida Tecnológico 2595, Fracc. Lagos del Country, Tepic 63175, Nayarit, Mexico; jumirodriguezji@ittepic.edu.mx (J.M.d.J.R.-J.); jragazzo@tepic.tecnm.mx (J.A.R.-S.)

**Keywords:** plant proteases, shrimp, by-products, hydrolyzates, bioactive peptides, antioxidant capacity

## Abstract

**Simple Summary:**

Guamara and cocuixtle fruits are underutilized and the need arises to use them because they can favor the production of hydrolyzates, in addition to the fact that white shrimp is the crustacean that is produced in the greatest quantity in Mexico, of which approximately 50% of the total weight of shrimp is wasted. The objective of this research was to determine the optimum conditions for obtaining protein hydrolyzates from cooked shrimp waste, since it has been mentioned that cooking the shells improves their digestibility. This study is of vital importance for the utilization of resources, in addition to opening the possibility of using a new drug with antioxidant capacity or, failing that, to be used as a food additive to produce functional foods.

**Abstract:**

Since the fruits of *Bromelia pinguin* and *Bromelia karatas* are rich in proteases, the aim of this research was to optimize the hydrolysis process of cooked white shrimp by-products due to the effect of these proteases. A robust Taguchi L_16’_ design was used to optimize the hydrolysis process. Similarly, the amino acid profile by GC-MS and antioxidant capacity (ABTS and FRAP) were determined. The optimal conditions for hydrolysis of cooked shrimp by-products were pH 8.0, 30 °C, 0.5 h, 1 g of substrate and 100 µg/mL of *B. karatas*, pH 7.5, 40 °C, 0.5 h, 0.5 g substrate and 100 µg/mL enzyme extract from *B. pinguin* and pH 7.0, 37 °C, 1 h, 1.5 g substrate and 100 µg/mL enzyme bromelain. The optimized hydrolyzates of *B. karatas B. pinguin* and bromelain had 8 essential amino acids in their composition. The evaluation of the antioxidant capacity of the hydrolyzates under optimal conditions showed more than 80% inhibition of in ABTS radical, *B. karatas* hydrolyzates had better higher ferric ion reduction capacity with 10.09 ± 0.02 mM TE/mL. Finally, the use of proteolytic extracts from *B. pinguin* and *B. karatas* to optimize hydrolysis process allowed obtaining hydrolyzates of cooked shrimp by-products with potential antioxidant capacity.

## 1. Introduction

Bromeliad fruits are considered rich in proteases and it is well known that they play an important physiological role in the development and mobilization of germ proteins in response to various types of environmental stress, defending against pests and pathogens, programmed cell death, and senescence of the fruits [1]. On the other hand, it has been reported that the proteases extracted from the fruits of these plants are effective for the production of protein hydrolyzates [2]. Moreno-Hernandez et al. [2] extracted pinguinain, which was obtained from *Bromelia pinguin* traditionally known as “guamara”. This enzyme has an optimal pH between 7.2–8.8, the optimal temperature is 35–50 °C, and a specific activity of 3.2 U/mg. The approximate molecular weight of the four protein components that make up this enzyme complex is 23 kDa [3,4]. On the other hand, the protease extracted from the fruit of *Bromelia karatas*, commonly kwon as “cocuixtle”, called karatasin, has a molecular weight of 28.86 kDa. It can be dissociated into active subunits, one with a molecular weight between 13 kDa and 3.5 kDa, while others with a molecular weight between 13 kDa and 20 kDa, when sodium dodecyl sulfate (SDS) and β-mercapto-ethanol are added [5]. Montalvo et al. [6] evaluated the physiological behavior and phytochemicals of guámara and cocuixtle, where they obtained a specific proteolytic activity in prepurified extracts of 53.88 U/mg of protein with the fruit in maturity type II (fruit ripe for consumption) for guámara and 18.99 U/mg of protein with fruit in maturity type I (green and firm fruit) for cocuixtle. These results confirmed that the isolated proteases had proteolytic activity. However, the highest activity was observed in *B. pinguin* proteases. In addition, the cytotoxic, anthelmintic and antimicrobial [3,6,7], anti-inflammatory [8] and antitumor [9] activities of the pre-purified extracts obtained from both fruits have also been studied [10,11]. Furthermore, it has been observed that they have shown remarkable thermal stability, as well as a high stability at high values of ionic strength (pI between 4.6 and 8.1) [3].

Recent applications of some of these proteases have been studied in the extraction of bioactive peptides [12]. These peptides are characterized by their important role in the regulation and modulation of human metabolism [12,13,14].

In 2017, the global production of farmed shrimp was 5716 billion tons in live weight [15], of which only 50–60% is considered to be due to the consumption of shrimp and the rest is discarded as a by-products (head and exoskeleton), where the head represents 30–45% of the total weight of the shrimp [15,16]. For the transformation of these residues, different alternatives have been sought with the intention of taking advantage of all the compounds present (protein, chitin, and astaxanthin) [17], for this reason some biological methods have been implemented, such as enzymatic hydrolysis through the use of proteases of plant origin. It has been previously reported that the sources of plant proteases from *B. pinguin* (guamara) and *B. karatas* (cocuixtle) allow the obtainment of low molecular weight peptides in animal by-products [18].

Bioactive peptides are short sequences ranging from 2 to 30 amino acids in length. It has been mentioned that the composition, the molecular weight (<1.35 kDa) and the order of the amino acid sequence, of the peptide defines the bioactivity that they might have [19,20]. Due to their high protein content, low or no toxicity and their potential impact on human health, bioactive peptides from marine byproducts have become increasingly important [20,21]. Therefore, enzymatic hydrolysis stands out as a good alternative for obtaining hydrolyzates from cooked shrimp by-products. It has also been found that sources of vegetable proteases, such as *B. pinguin* and *B. karatas*, have a high proteolytic activity [6,21] which allows the obtainment of peptides with biological activity and a low molecular weight. The aim of this research is to obtain the optimal conditions for the hydrolysis of cooked shrimp by-products and to obtain the amino acid profile of the hydrolyzates under the optimal conditions of enzymatic hydrolysis with proteolytic extracts of *B. pinguin*, *B. karatas*, and commercial Bromelain.

## 2. Materials and Methods

### 2.1. Biological Material

Raw shrimp by-products (the head and exoskeleton) were collected from a seafood restaurant in the city of Tepic, Nayarit, Mexico. The organic material was packaged in polyethylene bags and transported to the Integral Food Research Laboratory of the Technological Institute of Tepic. By-products were subjected to a heat treatment at 100 °C for 5 min, then they were frozen at −80 °C as a procedure prior to lyophilization (Free Zone 2.5 L −50 °C, LABCONCO, Kansas City, MI, USA) and size reduction of the by-products.

To obtain proteolytic extracts from *B. pinguin* and *B. karatas*, the methodology described by Romero-Garay et al. (2020) [12] was used. 10 g of lyophilized pulp were homogenized in a mixer (Tissue tearor 9853720, BIOSPEC products, INC, Bartlesville, OK, USA) with 100 mL of 0.1 M monobasic monohydrate sodium phosphate buffer (4 °C, pH 6.0, EDTA 5 mM and L-cysteine 5 mM) for 20 min and then centrifuged at 13,000 rpm for 15 min. The protein concentration and proteolytic activity of the supernatant were determined. To the crude extract obtained from cocuixtle proteases was added 20% (*w*/*v*) ammonium sulfate and to the crude extract of guamara 30% (*w*/*v*), respectively. It was homogenized for 10 min. It was left to stand for 4 h at 4 °C with constant agitation (Colosquid, IKA Works, INC, Wilmington, DC, USA), then centrifuged (HermLe, Z32HK, Labortechnik GmbH, Wehingen, Germany) at 13,000 rpm for 15 min at 4 °C. The supernatant was discarded to use the precipitate that was obtained during centrifugation. A dialysis membrane (cellulose, MWCO 13 kDa) was used to remove excess ammonium sulfate. Dialysis was carried out at 4 °C for 20 h with constant stirring (Colosquid, IKA Works, INC., Wilmington, NC, USA).

### 2.2. Optimal Hydrolysis Conditions under a Taguchi L_16’_ Design

A robust Taguchi L_16′_ design (Table 1) was used. This design was obtained with the STATISTICA v. 12 (StatSoft, Tulsa, OK, USA), which listed 16 treatments to evaluate 5 factors (pH, temperature, hydrolysis time, amount of substrate, and amount of enzyme) that were randomly combined. This design was applied to the proteolytic extracts of *B. karatas*, *B. pinguin*, and Bromelain (B4882-Bromelain from pineapple stem, Sigma-Aldrich^®^ (St. Louis, MO, USA) as a positive control. Using Bromelain, the hydrolysis processes were carried out under the following conditions: pH 7.0, 37 °C (as they are the optimal ones reported for this enzyme) [22]. The use of this experimental design allowed to evaluate the effect of each one of the factors with the extracts proposed in this investigation, to finally obtain the optimal hydrolysis parameters for each enzyme using the best level of each factor based on the response variables degree of hydrolysis, antioxidant activity against inhibition of 2,2′-Azino-bis-(3-ethylbenzothiazoline-6-sulphonic Acid Cation Radical (ABTS), and iron-reducing activity.

#### 2.2.1. Enzymatic Hydrolysis

The conditions of the treatments were taken into account in the design of Taguchi L_16′_ to carry out the enzymatic hydrolysis, for which the methodology established by Romero-Garay et al. was used. [18]. 30 mL of 0.2 M phosphate buffer (pH corresponding to each treatment) was added to which the corresponding amount of substrate (cooked lyophilized shrimp by-product) and placed for 15 min in a boiling water bath in order to deactivate the enzymes present in the shrimp head. These types of proteases are generally active at acid pH, increasing their activity at neutral or slightly alkaline pH [23]. Therefore, it is essential to mention that in this research, prior to enzymatic hydrolysis, the enzymes present in the shrimp by-products were deactivated at 98 °C for 15 min, thus ensuring that the effect of the degree of hydrolysis was due to the proteolytic extracts of *B. karatas* and *B. pinguin*. Subsequently, they were cooled at 4 °C for 10 min. Next, 1 mL of enzyme solution was added at the appropriate concentration for each treatment, mixed in a vortex (Genie 2, Scientific industries^TM^, New York, NY, USA) and the hydrolysis tubes were placed in an incubator (HCM-D59, Equip and technology systems S.A. de C.V., Nezahualcóyotl, México) at the corresponding temperature and time, with constant stirring. The hydrolyzates from each treatment were filtered through a gauze of approximately 2 mm, then heated in a boiling water bath for 15 min. The hydrolyzates were centrifuged (14,000 rpm for 10 min) (HermLe, Z32HK, Labortechnik GmbH, Wehingen, Germany). The supernatant was microfiltered with a 0.45 µm membrane filter (Millipore, MF-Membrane Filters HAWP, Merck Millipore, Darmstadt, Germany). Once the hydrolyzates were microfiltered, they were frozen at −80 °C for subsequent lyophilization (Free Zone 2.5 L–50 °C, LABCONCO, Kansas City, MI, USA) and storage at −20 °C until they were used.

#### 2.2.2. Antioxidant Capacity

##### Inhibition of 2,2′-Azino-bis-(3-ethylbenzothiazoline-6-sulphonic Acid Cation Radical (ABTS)

The antioxidant capacity was evaluated according to the method proposed by Re et al. [24]. A solution of 7 mM ABTS (A1888-2,2′ azinobis (3-ethylbenzothiazoline-6-sulfonic acid) diammonium salt, Sigma-Aldrich^®^) was prepared in sodium phosphate buffer (0.1 M, pH 7.4) with persulfate. potassium (2.45 mM), which was incubated with constant shaking (Colosquid, IKA Works, INC, Wilmington, DC, USA) at room temperature for 16 h, and the solution was subsequently adjusted to an absorbance of 0.7. Subsequently, 265 µL of ABTS solution and 35 µL of peptides (30 mg/mL) were placed in a microplate, left to rest for 10 min in the dark with constant shaking (4025, LAB LINE INSTRUMENTS Inc., Melrose Park, IL, USA). The absorbance was read at a wavelength of 730 nm in an 800 TS microplate reader (Biotek, Charlotte, VT, USA). Phosphate buffer and Trolox (238813-(±) 6-hydroxy-2,5,7,8-tetramethyl-chromane-2-carboxylic acid, Sigma-Aldrich^®^) were used as blank in different concentrations (32.5 µM to 600 µM) as a standard solution for carrying out a calibration curve. Results were expressed as percent inhibition.

##### Ferric Reducing Antioxidant Power (FRAP)

The ability of the peptides to reduce the ferric ion (Fe^3+^) to its ferrous ion (Fe^2+^) was evaluated by the method of Benzie & Strain [25]. The FRAP reagent was prepared from a sodium acetate buffer (300 mM, pH 3.6) solution of TPTZ ((2,4,6-tris(2-pyridyl)-s-triazine) solution in 40 mM of HCl) and FeCl_3_ (20 mM) in a 10:1:1 ratio and incubated (HCM-D59, Equip and Technology Systems, S.A. de C.V., Nezahualcóyotl, México) at 37 °C for 10 min in the dark. To begin the assay, 27 µL of peptides (30 mg/mL) with 230 µL of FRAP reagent and 23 µL of distilled water were added to a microplate, which was incubated at 37 °C for 30 min in the dark with constant shaking. Absorbance was read at 595 nm in an 800 TS microplate reader (Biotek). Phosphate buffer was used as blank and Trolox (238813-(±) 6-hydroxy-2,5,7,8-tetramethyl-chromane-2-carboxylic acid, Sigma-Aldrich^®^) as standard solution to perform a calibration curve at different concentrations (0.008125 to 0.13 mM). The results were expressed in mM TE/mL.

#### 2.2.3. Degree of Hydrolysis (DH)

The methodology proposed by Adler-Nissen [26] was used. In this research, a modification was made in the chromophore concentration, which was adjusted to a final volume of 4.120 mL. For this determination, 0.120 mL of hydrolyzate was mixed with 1 mL of buffered sodium phosphate (0.2125 M, pH 8.2) and 1 mL of 0.0125% (*v*/*v*) TNBS (2,4,6-trinitrobenzene-Thermo Scientific^TM^ sulfonic acid). The mixture was stored in an incubator (HCM-D59, Equip and Technology Systems, S.A. de C.V., Nezahualcóyotl, México) at 50 °C, for 60 min in the dark. The reaction was terminated by adding 2 mL of 0.1 N HCL, for 30 min in the dark at room temperature. Absorbance was measured in a spectrophotometer (Jenway 6705, Bibby Scientific Ltd., Dunmow, Essex, UK) at a wavelength of 340 nm. The blank was prepared by replacing the sample volume with an equal volume of monobasic sodium phosphate buffer (0.2125 M, pH 8.2). A calibration curve of L-leucine (L8000-L-Leucine, Sigma-Aldrich^®^) was prepared at concentrations from 0.1 mM to 2.5 mM. The results were expressed as a percentage of degree of hydrolysis according to the following formula:

$$DH\left(\%\right)=\frac{\left(NH_{2t}-NH_{0}\right)}{\left(NH_{2max}-NH_{0}\right)}\times 100$$

where *NH*_2*t*_ are the mmols before after hydrolyzing the by-products, *NH*_0_ are the mmols of unhydrolyzed by-products, and *NH*_2*max*_ are the mmols of the acid hydrolysis with 6N HCL for 24 h.

### 2.3. Aminoacid Profile by GC-MS under Optimal Hydrolysis Conditions

Amino acid determination was performed following the protocol proposed by Brion-Espinoza et al. [27]. Samples of optimized hydrolyzates of cooked shrimp by-products from different proteolytic extracts (*B. karatas*, *B. pinguin*, and bromelain) were subjected to acid hydrolysis with 6 M HCl + 0.06% (*w*/*v*) phenol for 24 h at 110 °C. Subsequently, the hydrolyzed samples were derivatized with MTBSTFA (N-tert-butyldimethylsilyl-N-methyltrifluoroacetamide). Once the samples were derivatized, 100 µL of hydrolyzate and 10 µL of L-Norleucine (N8513, L-Norleucine, Sigma-Aldrich^®^) were taken as an internal standard at a concentration of 0.2 mg/mL and were evaporated with nitrogen gas until dryness. The resulting precipitate was dissolved in 200 µL of acetonitrile and 200 µL of MTBSTFA (77626-N-tert-Butyldimethylsilyl-N-Methyltrifuoroacetamide for GC derivatization, LiChropurTM, Sigma-Aldrich^®^). This solution was incubated at 100 °C for 2.5 h in a glycerol bath. The derivatization reaction was performed in a 2 mL PTFE-lined screw cap vial. For the standard mixture of L-amino acids the same procedure was followed. Gas chromatography-mass spectrometry (GC-MS) analysis was performed using a 7890A GC (Agilent Technologies; Palo Alto, CA, USA) coupled to an MS 240 Ion Trap (Agilent Technologies; Palo Alto, CA, USA). An Agilent J&W VF-5ms capillary column (30 m × 0.25 mm, id, 0.25 μm film thickness) was used for amino acid separation. The carrier gas was Helium (99.99%) at a flow rate of 2 mL/min. The oven temperature program was set at 150 °C for 2 min, increased at 3 °C/min to 280 °C. 2 μL were injected with an autosampler in split mode (20:1) into the GC injector port at 260 °C. MS parameters were as follows: ionization energy (70 eV), full scan mode (35–650 *m*/*z*), ion trap (150 °C), collector (80 °C), and transfer line (130 °C). Linear retention indices were calculated using a mixture of straight-chain alkanes (C7–C30), injected under the same assay conditions. The amino acid profile was reported as g amino acid/100 g protein.

### 2.4. Statistical Analysis

As already mentioned, the project was developed under a Taguchi design, therefore, under this design, the data were analyzed (STATISTICA v. 12 StatSoft, Tulsa, OK, USA).

## 3. Results

### 3.1. Antioxidant Capacity

#### 3.1.1. Inhibition of 2,2′-Azino-bis-(3-ethylbenzothiazoline-6-sulphonic Acid Cation Radical (ABTS)

It can be observed that the inhibition percentage varies according to the treatments that were carried out according to the Taguchi L_16′_ Design (Table 2). The Taguchi L_16′_ analysis showed that factors including pH, temperature, the amount of substrate, and the amount of enzyme were significant (*p* < 0.05) in determining the antioxidant capacity when using the ABTS method for the hydrolyzates obtained with proteolytic extracts of *B. karatas*. Only the pH and temperature were significant (*p* < 0.05) in the hydrolyzates obtained with proteolytic extracts of *B. pinguin*.

##### 3.1.2. Ferric Reducing Antioxidant Power

The variation between the treatments performed according to the Taguchi L_16′_ design and their antioxidant capacity can be observed (Table 3). The Taguchi L_16′_ analysis showed that in the hydrolyzates obtained from *B. karatas* proteolytic extracts, factors including the pH, temperature, and the amount of substrate were significant (*p* < 0.05). For the hydrolyzates obtained from *B. pinguin* proteolytic extracts, only temperature had an effect (*p* < 0.05). For the hydrolyzates obtained from bromelain proteolytic extracts, only the amount of enzyme had a significant effect (*p* < 0.05).

### 3.2. Degree of Hydrolysis

According to the Taguchi L_16′_ design, variations in the percentage of the degree of hydrolysis can be observed between the treatments (Table 4). It should be noted that all the variables were statistically significant (*p* < 0.05) for the proteolytic extracts of *B. karatas* and *B. pinguin*.

In order to select the optimal levels of each factor for each proteolytic extract in the STATISTICA v.12 program, the results obtained from the response variables of antioxidant capacity and degree of hydrolysis from the previous sections were used as a database. The following criteria were used to select the factor levels under optimal hydrolysis conditions. They are listed below:

- The minimum level set by the STATISTICA program is used if the factor is not significant.
- If only one variable in the factor is significant, this is considered as the optimal condition.

The optimal levels selected for each proteolytic extract are shown below.

### 3.3. Analysis of Hydrolysates Obtained under Optimal Hydrolysis Conditions with Proteolytic Extracts of B. pinguin, B. karatas and Bromelain

#### 3.3.1. Selection of Optimal Hydrolysis Conditions with Proteolytic Extract of *B. pinguin*

**Figure 1:**
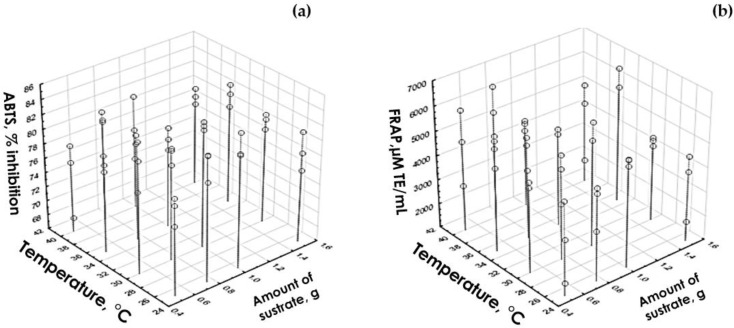
Graph to determine the optimal level of the temperature factor in hydrolyzates with *B. pinguin* response variable: (**a**) ABTS and (**b**) FRAP.

To determine the optimal level of the five factors shown in Table 5, the variables corresponding to antioxidant capacity (ABTS and FRAP) were considered. The optimal level of the factors pH, hydrolysis time, and the amount of substrate was taken in accordance with the level that coincided in ABTS and FRAP. The amount of enzyme was not significant for the response variables of antioxidant capacity. Therefore, it was decided to use the 100 µg/mL level because this level had an effect on the degree of hydrolysis variable of degree of hydrolysis. In order to determine the optimal temperature level, it was necessary to create a graph with three axes (Figure 1). The condition that favors the response variable was chosen, since in both cases there were significant differences.

In Figure 1a, it can be seen that for ABTS there is a slight difference between the levels corresponding to 30 and 35 °C (corresponding to levels 2 and 3 of this factor) in the optimal amount of substrate (1 g), being higher the percentage of inhibition at 35 °C, in the same way in FRAP (Figure 1b) the difference between the 2 temperature levels to be selected (2 and 3) can be noted, where the highest level is at a temperature of 30 °C when there is an optimal amount of substrate (1 g). For this reason, level 2 was selected. This corresponds to 30 °C.

#### 3.3.2. Selection of Hydrolyzates under Optimal Conditions with Proteolytic Extract of *B. karatas*

The results of the optimal hydrolysis conditions using proteolytic extracts of *B. karatas* are shown in Table 6. To select the optimal level of the pH, enzyme amount, and substrate amount, the level that showed an effect on both ABTS and FRAP was considered. For the selection of the temperature, the level of the ABTS variable was taken into account. For the hydrolysis time, the level of the FRAP variable was taken into account, since these were the ones that showed a significant effect.

#### 3.3.3. Selection of Hydrolyzates under Optimal Conditions with Bromelain

In this case, the selection of the optimal conditions for the reagent grade bromelain enzyme was easier because Hernandez et al. [23] had already defined an optimum pH and temperature, so only the optimal conditions were selected for the factors hydrolysis time, amount of substrate, and amount of enzyme (Table 7).

### 3.4. Antioxidant Capacity under Optimal Hydrolysis Conditions

#### 3.4.1. Inhibition of 2,2′-Azino-bis-(3-ethylbenzothiazoline-6-sulphonic Acid Cation Radical under Optimal Hydrolysis Conditions

**Figure 2:**
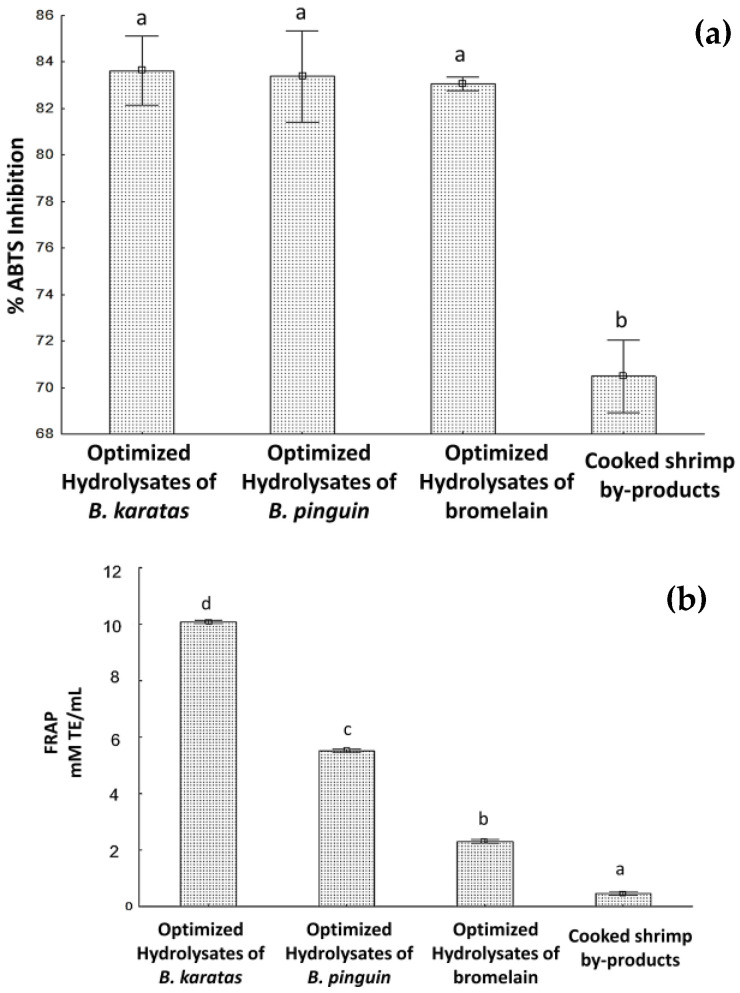

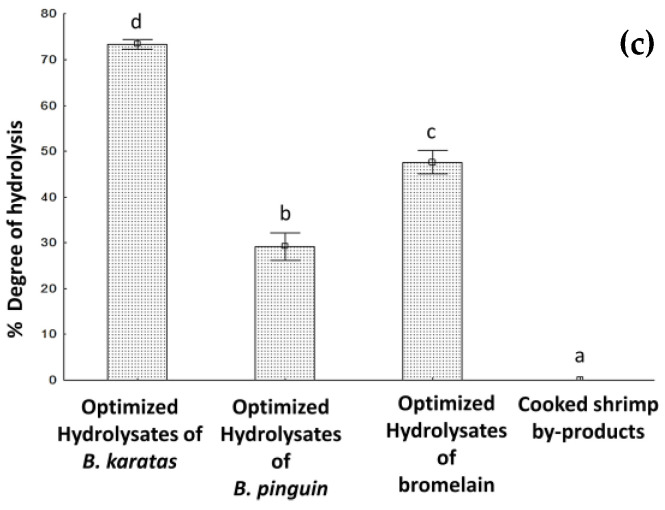
Optimized hydrolyzates results: (**a**) ABTS (**b**) FRAP (**c**) Degree of hydrolysis. Different letters signify significant differences (one-way ANOVA, Fisher LSD, *p* < 0.05).

Figure 2a shows the results obtained from the percentages of inhibition of the ABTS^•+^ radical. It can be seen that there are no statistically significant differences between the optimized hydrolyzates of *B. karatas* (83.6 ± 0.47%), *B. pinguin* (83.5 ± 1.4%) and Bromelain (83 ± 0.1%), compared to that obtained for cooked shrimp by-products (70 ± 0.8%), which did have significant differences with respect to the hydrolyzates. With this information it was possible to elucidate that the determination of the optimal hydrolysis conditions (pH, temperature, hydrolysis time, amount of substrate, and amount of enzyme) allowed the enzymes involved in the hydrolysis process to act on the cooked shrimp by-products, modifying its protein structure, which presented better antioxidant capacity to inhibit the, ABTS radical with respect to non-hydrolyzed by-products.

#### 3.4.2. Ferric Reducing Antioxidant Power under Optimal Hydrolysis Conditions

The results of the measurement of antioxidant capacity by the FRAP method for hydrolyzates under optimal hydrolysis conditions and cooked shrimp by-products are shown in Figure 2b. A significant difference can be observed in all the hydrolyzates that were evaluated. In the same way, it is observed that the hydrolyzates have a better capacity to reduce the ferric ion (Fe^3+^) to its ferrous form (Fe^2+^) (by the donation of an electron or hydrogen) than unhydrolyzed by-products.

### 3.5. Degree of Hydrolysis under Optimal Hydrolysis Conditions

Figure 2c shows the results obtained for the 3 optimized hydrolyzates and the by-products of cooked shrimp without hydrolysing. It is shown that there were statistically significant differences for all hydrolyzates under optimal hydrolysis conditions. The hydrolyzate optimized with *B. karatas* had 73.3 ± 0.4% DH, followed by the hydrolyzates optimized with Bromelain (47.6 ± 1.0%) and *B. pinguin* (29.2 ± 1.2%).

### 3.6. Amino Acid Profile by GC-MS under Optimal Hydrolysis Conditions

The results obtained from the amino acid profile using GC-MS for their quantification are shown in Table 8. The amino acids were identified by comparing their retention times with the standard amino acids in specific MS chromatograms; the quantitative analysis was performed using L-norleucine as an internal standard (0.2 mg/mL).

## 4. Discussion

### 4.1. Antioxidant Capacity

#### 4.1.1. Inhibition of 2,2′-Azino-bis-(3-ethylbenzothiazoline-6-sulphonic Acid Cation Radical

pH can affect the dynamics of association of the active site of the enzyme with the substrate present in the reaction, by causing structural changes in the enzyme or slowing the rate of hydrolysis reaction. Therefore, the antioxidant capacity is also affected by this factor [28,29]. The different percentage of inhibition between *B. karatas* and *B. pinguin* in terms of the significant values of the factors used in the experimental design could be explained because they have different proteolytic activities. In the case of *B. pinguin*, the proteolytic activity of this enzyme is more than double with respect to that of *B. karatas*, which could be reflected in the percentages of inhibition to the ABTS radical, with *B. pinguin* having the highest percentages in all the treatments of the experimental design. For bromelain, only the amount of substrate was significant. As mentioned in the previous section, a change in the amount of substrate can positively or negatively affect the enzyme, even inhibiting it if the substrate exceeds the amount to be applied [30]. It is important to highlight that there are few reports that exist in relation to cooked shrimp by-products; Wan et al. [31] identified and characterized an antioxidant peptide from chicken feather keratin hydrolyzates, reporting 80% inhibition by the ABTS method using Bacillus subtilis for hydrolysis, employing 72 h and 37 °C with a concentration of these by-products of ~0.35 mg/mL. These authors argue that its greater antioxidant capacity was due to the high amount of hydrophobic amino acids, since peptides can bind to or cross the cell membrane, which facilitates access to free radicals; therefore, they can act by preventing the oxidation of the cell membrane mediated by peroxyl radicals or by inhibiting intracellular oxidation. In addition, it has been related that the elimination of radicals can be produced by the donation of hydrogen atoms [32].

It should be noted that the kinetics and mechanism of action between ABTS radical cations and antioxidant peptides have not been reported [33]. The antioxidant capacity of a peptide can be described by the composition of amino acids, molecular weight, and the sequence of amino acids in the peptide, such is the case of the aromatic amino acids (AAA) (Phe, Tyr and Trp) which have shown that when found in the terminal chain of the peptide, exert greater antioxidant capacity in addition to having the ability to form H-bridges [32], on the other hand, the electronic and hydrophobic properties of the C and N terminal amino acids have also been correlated with antioxidant capacity [34].

The percentages of inhibition in the hydrolyzates of cooked shrimp by-products can be attributed to hydrophobic amino acids (HAAs) (62.8–70.4%). These can act as proton donors or electron scavengers of free radicals [35]. In addition, the presence of AAA in the terminal chain of the peptide has been attributed to its ability to neutralize free radicals. It acts as an electron/hydrogen donor due to the presence of indole, imidazole and phenolic groups [36,37]. In order to make a connection with what was said before, it would be necessary to carry out an amino acid sequencing of the obtained hydrolyzates. However, with the information obtained from the determination of the amino acid profile, it can be mentioned that there was a 5.2–7.9% of this group of amino acids, so it can be concluded that there can be a positive effect of these amino acids on the antioxidant capacity by this method.

#### 4.1.2. Ferric Reducing Antioxidant Power

Nikoo et al. [38] reported the antioxidant capacity of white shrimp by-products by means of autolysis at a time of 1 h, a temperature of 40 °C, and a pH of 8.0, obtaining 65 ± 0.1 µM TE/g. It is worth mentioning that high concentrations of low molecular weight peptides present in hydrolyzates favor donating electrons to free radicals [39], which translates into increased antioxidant capacity. As can be seen, the hydrolysis conditions used in the study by Nikoo et al. [38] are similar to the levels of each factor that were used in the experimental design; however, the difference was the hydrolysis method, so with these results it was possible to confirm that an enzymatic hydrolysis with cysteine proteases confers a greater antioxidant capacity to hydrolyzates from cooked shrimp by-products. In general, it has been mentioned that the antioxidant capacity is attributed to peptides that have a low molecular weight, so it has been correlated in some way with the degree of hydrolysis, which has been widely used as an indirect tool to describe the antioxidant capacity. The distribution of the molecular weight of hydrolyzates, so that a prolonged hydrolysis almost always results in high degrees of hydrolysis and therefore much smaller peptide chains are obtained [40]. There is a global trend to increase the antioxidant capacity in vitro as hydrolysis progresses, with large differences in the concentrations of FRAP and ABTS [41]. However, comparing all this information with the results obtained, it can be commented that it cannot yet be directly related to the degree of hydrolysis. with the antioxidant capacity obtained, since in some treatments there was a high degree of hydrolysis and low antioxidant capacity which could indicate that the amino acid composition has an important repercussion to be able to define if there is any relationship between both determinations. From the results obtained, it is concluded that the mechanism of action of the hydrolyzates obtained is that of a metal chelating agent.

Only amino acid composition was determined in this study. The optimized hydrolyzate of *B. karatas* (10.09 ± 0.02 mM TE/mL) has a higher metal chelating capacity. This is because it is the hydrolyzate with the highest amount of Glu (9.8 g /100 g protein), a good amount of AAA (7.5%) and one of HAAs (70.4%), compared to the other hydrolyzates studied. This has been shown to have a positive effect on the antioxidant capacity by this method [42,43]. Thus, it could be concluded that these groups of amino acids could chelate metals by electron or hydrogen donation due to the presence of imidazole, indole, and phenolic groups [44].

It is very important to emphasize that even the bromelain optimized hydrolyzate with 23.1 ± 0.3 mM ET/mL, which is considered the lowest of the three hydrolyzates studied (AAA: 7.8%, HAAs: 57.1% and Glu: 17.6 g/100 g protein) and has a higher antioxidant capacity than the one obtained by Dayakar et al. [45].

In this study of antioxidant capacity by both methods (ABTS and FRAP) it was possible to demonstrate that the optimized hydrolyzates obtained from cooked shrimp by-products using the optimized hydrolyzates with *B. karatas*, *B. pinguin* and Bromelain have antioxidant capacity (ABTS and FRAP), could have a health benefit. Based on this study, it would be interesting to evaluate what other types of bioactivities (could be exhibited by these optimized hydrolyzates.

### 4.2. Degree of Hydrolysis

According to the Taguchi L_16′_, all the variables were significant (*p* < 0.05) in the hydrolyzates obtained with proteolytic extracts of *B. karatas* and *B. pinguin*. Therefore, the hydrolyzates show different degrees of hydrolysis. The pH factor is related to the stability of the enzyme, since a pH outside the optimum range of the enzyme can slow down the hydrolysis. In addition, a pH that is very different from the optimum for each enzyme can modify its conformational structure, resulting in a decrease in the degree of hydrolysis [28,29]. On the other hand, the use of high temperatures in enzymatic hydrolysis can exert a decrease in the degree of hydrolysis because high temperatures promote conformational denaturation of the enzyme, however, a low temperature could increase the hydrolysis time [46]. The interaction of the factors of amount of enzyme and amount of substrate was described by Michaelis and Menten) [47] where they postulated that the speed of enzyme activity depends on the concentration of substrate and enzyme used, since an increase in enzyme concentration it can speed up hydrolysis, as long as there is available substrate to bind to, so once all the substrate is bound to the enzymes the reaction reaches its maximum speed [46,48].

For bromelain, which was used as a control, only the amount of substrate was significant. As the amount of substrate is modified, there may be a decrease in the concentration of peptide bonds susceptible to hydrolysis by the action of proteases or a possible inhibition of enzymes caused by the amount of substrate [30]. It is important to highlight that the shrimp hepatopancreas is an important source of proteases such as trypsin and chymotrypsin [16]. These types of proteases are generally in acidic a pH, increasing their activity at a neutral or slightly alkaline pH [49]; For this reason, it is essential to mention that, in this investigation, prior to the enzymatic hydrolysis, an inactivation of the enzymes present in the shrimp by-products was carried out at 98 °C for 15 min. This ensured that the effect of the degree of hydrolysis was due to the proteolytic extracts of *B. karatas* and *B. pinguin*.

There are some studies in which the degree of hydrolysis of by-products and shrimp cooking water was evaluated. Tonon et al. [46] obtained 16–48% degree of hydrolysis in shrimp cooking water using 2.4 L alcalase at a temperature of 75 °C, pH 9.0 and E/S ratio of 0.1%. As can be seen, the results obtained by Tonon et al. [46] are different from those obtained in this research due to the type of substrate that was used in the enzymatic hydrolysis, and the hydrolysis conditions were different, in addition to the fact that the temperature used in this study was lower than that of Tonon et al. [4]. Ketnawa et al. [50] obtained hydrolyzates from whieleg shrimp (*Penaeus vannamei*); these hydrolyzates had the highest DH with alcalase (5.49%). In addition, Djellouli et al. [32] obtained protein hydrolyzates from cooked white shrimp by-products and shrimp cooking water using an enzyme extract from *Enterococcus faecalis* DM19 isolated from raw camel milk, at a pH of 7.0 and a temperature of 40 °C per two hours of incubation, where its degree of hydrolysis for the by-products was 12.60% and for cooking water 9.89%, respectively. When comparing the hydrolysis conditions used in this study, similarities can be observed in the hydrolysis parameters (substrate, pH, temperature, and the time of incubation). However, the use of different enzymatic extracts was apparently the determining factor in the degree of hydrolysis obtained by Djellouli et al. [32]. From the information provided by other researchers and the results obtained in this study, it can be concluded that proteolytic extracts from Bromeliaceae plants have an affinity for the shrimp by-product substrate, since they provide a better degree of hydrolysis than other enzymes (such as alcalase 2.4 L and proteolytic extracts of Enterococcus faecalis DM19), even when all the hydrolysis conditions are modified (amount of substrate, amount of enzyme, pH value, hydrolysis time, and temperature).

The hydrolyzate optimized with *B. karatas* obtained the best metal chelating capacity (10.09 ± 2.4 mM TE/mL) coinciding with its DH being the highest. On the other hand, Romero-Garay et al. [50], in proteolytic extracts obtained from the same plants and using the same protease extraction procedure [4], evaluated the DH in chicken and fish by-products and reported 2.2–22.1% of DH. They attributed that a DH greater than 10% could generate peptides with high solubility, while a DH of 1–10% could improve the functional properties (foaming and emulsifying) in food products [51]. 

In another investigation, Meza-Espinoza et al. [52] evaluated the DH of *B. karatas*, *B. pinguin* and Bromelain in different sources of protein concentrates (soybean, milk, and ovalbumin), and obtained a high DH for *B. karatas* (49.85 ± 1.63–73.88 ± 2.95%), *B. pinguin* (65.71 ± 2.49–81.74 ± 1.74%) and Bromelain (55.53 ± 0.59–76.71 ± 0.62%) in the different types of substrates used, however, the highest DH was obtained in soybean protein. The results obtained from the DH in by-products of cooked shrimp were similar in the hydrolyzates optimized with *B. karatas*, however, for the other optimized hydrolyzates there was a decrease in DH compared to that reported by Meza-Espinoza et al. [52]. These differences can be mainly attributed to the type of substrate used.

### 4.3. Amino Acid Profile by GC-MS under Optimal Conditions

The amino acids Lys, Arg, and His could not be identified. This is probably due to incomplete derivatization and/or formation of by-products from silylation of the compounds with MTBSTFA. These by-products could interfere with their quantification [53]. Furthermore, in none of the samples could the amino acid Trp derived from the hydrolysis process be quantified. This is because the side chain of the indole group is destroyed by oxidation under acidic conditions. This makes it impossible to derivatize the amino acid [54]. Regarding the amino acid profiles obtained, it can be noted that the optimized hydrolyzates of *B. karatas* have a higher amino acid content than unhydrolyzed cooked shrimp by-products. Regarding the amount of essential amino acids, the optimized hydrolyzates of *B. karatas*, *B. pinguin*, and bromelain had 8 out of 9 amino acids, as did the cooked shrimp by-products. There is no report of amino acid quantification of protein hydrolyzates of cooked white shrimp by-products by GC-MS, however, there is a report of amino acid quantification in HPLC using L-norleucine as an internal standard to know the amino acid profile of shrimp by-product hydrolyzates by Ambigaipalan et al. [55] where they reported that in general the hydrolyzates they obtained had amino acid profiles very similar to those of this research. In these, only the amounts of amino acids (g/100 g of protein) varied, which could be due to the specificity of the endopeptidase alcalase [56]. In the same way, the predominant amino acids in the hydrolyzates of Ambigaipalan et al. [55] were His and Ala. These results do not coincide because the predominant amino acids for the cooked shrimp by-products and the hydrolyzates were Asp and Glu. The differences may be due to the use of Trypsin in the enzymatic hydrolysis since it cleaves the C side chains of basic amino acids, such as His, and therefore acts significantly on aliphatic amino acids such as Ala [20,21]. In this research, pre-purified proteolytic extracts were used (for which there is no established hydrolysis mechanism). However, it is inferred that there is a consortium endo- and exo-peptidases which can act on the N and C terminal chain of cooked shrimp by-product proteins, resulting in a large amount of acidic amino acids, such is the case of Asp and Glu [57].

## 5. Conclusions

Protein hydrolysis is an effective method to revalorize cooked shrimp by-products. The optimal conditions to hydrolyze cooked shrimp by-products were pH 8.0, 30 °C, 0.5 h, 1 g substrate and 100 µg/mL proteolytic extract of *B. karatas*, pH 7.5, 40 °C, 0.5 h, 0.5 g substrate and 100 µg/mL proteolytic extract of *B. pinguin*, and pH 7.0, 37 °C, 1 h, 1.5 g substrate and 100 µg/mL bromelain enzyme. The results of this study indicate that enzymatic hydrolysis of cooked shrimp by-products is a good method to obtain hydrolyzates with antioxidant capacity with each proteolytic extract used (*B. karatas*, *B. pinguin*, and bromelain), highlighting that the hydrolyzates obtained with proteolytic extracts of vegetable origin (*B. karatas* and *B. pinguin*) presented a greater antioxidant effect than with a commercial enzyme. On the other hand, it is important to highlight that the amino acid profile obtained for the 3 hydrolyzates under optimal conditions presented a good amount of essential amino acids (Ile, Leu, Thr, Val, Met, Phe and Tyr). Further studies are needed to obtain more information on shrimp by-product hydrolyzates cooked under optimal hydrolysis conditions.

## Figures and Tables

**Table 1:** Experimental design of the robust Taguchi L_16′_ design.

Treatment	Factors				
	pH	Temperature, °C	Time, h	Substrate, g	Enzyme, µg/mL
1	6.5	25	0.5	0.5	50
2	6.5	30	1	0.75	100
3	6.5	35	2	1	150
4	6.5	40	4	1.5	250
5	7.0	25	1	1.5	150
6	7.0	30	0.5	1	250
7	7.0	35	4	0.75	50
8	7.0	40	2	0.5	100
9	7.5	25	2	0.75	250
10	7.5	30	4	0.5	150
11	7.5	35	0.5	1.5	100
12	7.5	40	1	1	50
13	8.0	25	4	1	100
14	8.0	30	2	1.5	50
15	8.0	35	1	0.5	250
16	8.0	40	0.5	0.75	150

**Table 2:** Antioxidant capacity when using the AABTS method in protein hydrolyzates of cooked shrimp by-products, ordered by the effect of the pH, temperature, time, the amount of substrate, and the amount of enzyme.

Treatment	ABTS, % Inhibition		
	*B. karatas*	*B. pinguin*	Bromelain
1	71.0 ± 1.1 ^ab^	77.7 ± 1.9 ^ab^	66.27 ± 2.4 ^a^
2	75.6 ± 0.9 ^cde^	78.6 ± 2.6 ^bcd^	76.9 ± 0.9 ^a^
3	76.5 ± 0.2 ^cde^	81.9 ± 2.0 ^cde^	77.0 ± 0.7 ^c^
4	82.5 ± 0.2 ^f^	82.5 ± 1.6 ^de^	77.0 ± 1.3 ^c^
5	78.3 ± 1.4 ^de^	80.6 ± 1.2 ^bcde^	66.4 ± 4.7 ^c^
6	77.5 ± 0.3 ^de^	82.7 ± 0.5 ^e^	76.4 ± 1.6 ^bc^
7	72.6 ± 0.1 ^ab^	82.2 ± 1.4 ^cde^	65.8 ± 3.6 ^bc^
8	73.5 ± 0.7 ^cd^	80.2 ± 1.0 ^bcde^	79.4 ± 0.3 ^c^
9	73.3 ± 0.6 ^bc^	78.3 ± 1.5 ^bc^	75.8 ± 3.8 ^c^
10	69.9 ± 0.3 ^a^	79.9 ± 0.8 ^ab^	77.0 ± 2.1 ^a^
11	79.5 ± 2.3 ^ef^	81.2 ± 1.5 ^bcde^	76.5 ± 3.5 ^c^
12	78.1 ± 1.0 ^de^	78.2 ± 1.1 ^bc^	65.8 ± 0.5 ^c^
13	76.1 ± 0.0 ^cd^	78.5 ± 1.0 ^bcd^	79.2 ± 1.5 ^c^
14	75.3 ± 0.5 ^cd^	76.9 ± 1.6 ^a^	76.4 ± 2.4 ^c^
15	77.0 ± 2.4 ^cde^	77.2 ± 4.3 ^ab^	78.5 ± 0.1 ^a^
16	79.2 ± 1.2 ^cde^	80.2 ± 0.7 ^bcde^	67.1 ± 0.8 ^ab^

Values are mean ± standard deviation (*n* = 3). Lowercase letters per row indicate a significant differences (*p* < 0.05) between hydrolysates obtained with different enzyme extracts and lowercase letters per columns indicate statistical differences (*p* < 0.05) between different conditions of treatment.

**Table 3:** Antioxidant capacity when using the FRAP method in protein hydrolyzates of cooked shrimp by-products by effect of pH, temperature, time, the amount of substrate, and the amount of enzyme.

Treatment	Ferric Reducing Antioxidant Power, mM TE/mL		
	*B. karatas*	*B. pinguin*	Bromelain
1	1.31 ± 0.04 ^ab^	4.06 ± 1.07 ^a^	0.23 ± 0.01 ^a^
2	1.46 ± 0.05 ^ab^	4.17 ± 0.44 ^ab^	1.48 ± 0.003 ^ab^
3	2.08 ± 0.05 ^bcde^	4.14 ± 1.23 ^abcd^	0.48 ± 0.07 ^cd^
4	2.77 ± 0.03 ^f^	5.31 ± 0.14 ^cde^	1.15 ± 0.04 ^f^
5	2.61 ± 0.17 ^ef^	5.00 ± 0.39 ^abcde^	0.65 ± 0.01 ^def^
6	2.76 ± 0.37 ^cde^	5.70 ± 0.51 ^de^	1.26 ± 0.003 ^cd^
7	1.43 ± 0.02 ^a^	4.59 ± 015 ^abcde^	0.45 ± 0.02 ^ab^
8	2.12 ± 0.15 ^bcde^	4.31 ± 0.08 ^abcd^	1.26 ± 0.01 ^a^
9	1.97 ± 0.04 ^abcd^	4.85 ± 0.11 ^bcde^	0.87 ± 0.02 ^a^
10	1.66 ± 0.14 ^abc^	5.01 ± 0.21 ^abcde^	0.74 ± 0.01 ^a^
11	2.58 ± 0.28 ^de^	5.99 ± 0.52 ^e^	1.35 ± 0.06 ^ef^
12	1.58 ± 0.05 ^abc^	5.44 ± 0.34 ^cde^	0.46 ± 0.11 ^cde^
13	1.82 ± 0.04 ^bd^	4.72 ± 0.53 ^abc^	1.82 ± 0.19 ^bc^
14	2.54 ± 0.21 ^e^	5.32 ± 0.89 ^abcde^	0.29 ± 0.06 ^cde^
15	1.68 ± 0.13 ^ab^	4.39 ± 0.28 ^abcd^	1.13 ± 0.007 ^a^
16	1.47 ± 0.22 ^abc^	5.34 ± 0.73 ^cde^	0.48 ± 105.0 ^ab^

Values are mean ± standard deviation (*n* = 3). Lowercase letters per row indicate significant differences (*p* < 0.05) between hydrolysates obtained with different enzyme extracts and lowercase letters per columns indicate statistical differences (*p* < 0.05) between different conditions of treatment.

**Table 4:** Degree of hydrolysis in protein hydrolyzates of cooked shrimp by-products by effect of pH, temperature, time, the amount of substrate, and the amount of enzyme.

Treatment	Degree of Hydrolysis		
	*B. karatas*	*B. pinguin*	Bromelain
1	25.4 ± 1.0 ^h^	15.8 ± 0.8 ^e^	20.9 ± 0.5 ^fg^
2	15.2 ± 1.0 ^e^	18.7 ± 0.7 ^f^	22.1 ± 1.2 ^g^
3	26.0 ± 0.2 ^h^	16.4 ± 0.6 ^ef^	3.0 ± 0.5 ^abcd^
4	12.6 ± 1.0 ^d^	6.2 ± 0.8 ^c^	3.2 ± 0.5 ^abc^
5	9.9 ± 1.1 ^c^	3.8 ± 0.8 ^b^	22.2 ± 1.4 ^ab^
6	16.7 ± 0.8 ^f^	25 ± 0.6 ^b^	2.2 ± 0.3 ^g^
7	6.4 ± 0.7 ^b^	10.2 ± 0.3 ^d^	91.9 ± 1.1 ^cd^
8	88.1 ± 0.5 ^i^	48.9 ± 1.4 ^h^	5.9 ± 0.7 ^k^
9	14.2 ± 0.8 ^e^	71.3 ± 1.3 ^j^	0.6 ± 0.1 ^f^
10	10.0 ± 0.4 ^c^	59.3 ± 3.0 ^i^	68.4 ± 1.7 ^i^
11	1.3 ± 0.1 ^a^	3.5 ± 0.6 ^ab^	6.1 ± 0.7 ^a^
12	19.1 ± 0.8 ^g^	16.0 ± 0.2 ^e^	64.3 ± 0.6 ^j^
13	1.1 ± 0.2 ^a^	0.7 ± 02 ^a^	14.7 ± 1.0 ^d^
14	14.4 ± 1.2 ^e^	3.6 ± 0.6 ^b^	21.7 ± 1.7 ^bcd^
15	26.3 ± 1.3 ^g^	3.1 ± 0.5 ^b^	4.9 ± 0.4 ^h^
16	12.1 ± 0.2 ^d^	41.8 ± 0.3 ^g^	18.4 ± 1.6 ^e^

Values are mean ± standard deviation (*n* = 3). Lowercase letters per row indicate significant differences (*p* < 0.05) between hydrolysates obtained with different enzyme extracts and lowercase letters per columns indicate statistical differences (*p* < 0.05) between different conditions of treatment.

**Table 5:** Optimum values obtained in the Taguchi L_16′_ analysis of the hydrolyzates using *B. pinguin*.

Factor	Degree of Hydrolysis	ABTS	FRAP	Optimum
pH	**8**	**8**	8	8
Temperature, °C	**35**	**30**	**35**	30
Hydrolysis time, h	0.5	0.5	0.5	0.5
Substrate amount, g	1	1	1	1
Enzyme amount, µg/mL	100	250	100	100

The values corresponding to significant factors (*p* < 0.05) are shown in bold font.

**Table 6:** Optimum values obtained in the Taguchi L_16′_ analysis of the hydrolyzates using *B. karatas*.

Factor	Degree of Hydrolysis	ABTS	FRAP	Optimum
pH	**8**	**7.5**	**7.5**	7.5
Temperature, °C	**40**	**40**	35	40
Hydrolysis time, h	**4**	1	**0.5**	0.5
Substrate amount, g	**1**	**0.5**	**0.5**	0.5
Enzyme amount, µg/mL	**50**	**100**	100	100

The values corresponding to significant factors (*p* < 0.05) are shown in bold font.

**Table 7:** Optimum values obtained in the Taguchi L_16′_ analysis of the hydrolyzates using bromelain.

Factor	Degree of Hydrolysis	ABTS	FRAP	Optimum
Hydrolysis time, h	1	1	4	1
Substrate amount, g	**0.5**	**1.5**	0.75	1.5
Enzyme amount, µg/mL	50	50	**100**	100

The values corresponding to significant factors (*p* < 0.05) are shown in bold font.

**Table 8:** Amino acid composition (g of amino acid/100g of protein) of cooked shrimp by-products and protein hydrolyzates of cooked shrimp by-products obtained with proteolytic extracts by GC-MS.

Amino Acids (g of Amino Acid/100 g of Protein)		GC-MS				Requirements According to the FAO (1991) (g of Amino Acid/100 g of Protein)	
		Shrimp By-Products	*B. karatas*	*B. pinguin*	Bromelain	Children	Adults
Essential	Isoleucine	8.8	9.9	7.9	7.3	2.8	1.3
	Leucine	13.9	20.5	13.8	12.4	6.6	1.9
	Lysine	ND	ND	ND	ND	5.8	1.6
	Tryptophan	ND	ND	ND	ND	1.1	0.5
	Histidine	ND	ND	ND	ND	1.9	1.6
	Threonine	4.3	5.4	7.1	6.4	1.4	0.9
	Valine	12.0	14.6	11.4	11.2	3.5	1.3
	Methionine ^1^	2.4	2.0	1.8	2.2	2.5	1.7
	Phenylalanine ^2^	7.9	8.9	9.5	11.3	6.3	1.9
Non-essential	Aspartic Acid	5.7	8.9	9.5	11.3		
	Glutamic acid	8.0	9.8	13.1	17.6		
	Serine	3.5	2.9	5.2	5.7		
	Glycin	10.5	2.5	2.2	2.0		
	Arginine	ND	ND	ND	ND		
	Alanin	14.0	11.2	14.8	11.5		
	Proline	8.7	4.7	8.0	4.6		
% Amino acid distribution	HAA	67.8	70.4	62.8	57.1		
	AAA	7.9	7.5	5.2	7.8		
	EAA	49.5	59.9	47.2	47.3		
	NCAA	21.7	27.1	34.9	41.0		
	PCAA	ND	ND	ND	ND		
	BCAAs	34.8	44.9	33.1	30.9		

HAA: Hydrophobic Amino Acids (Ala, Val, Ile, Leu, Tyr, Phe, Trp, Pro, Met, Cys) AAA: Aromatic Amino Acids (Phe, Trp, Tyr), EAA: Essential Amino Acids (His, Ile, Leu, Lys, Met, Phe, Thr, Trp, Val, Tyr, Cys) NCAA: Negatively charged amino acids (Asp, Arg, Glu, Thr, Ser) PCAA: Positively charged amino acids (Arg, His, Lys) BCAAs: Branched-chain amino acids (Ile, Leu, Val). ND: Not detected. ^1^ Methionine + Cysteine, ^2^ Phenylalanine + Tyrosine.

## Data Availability

The data generated from the study are clearly presented and discussed in the manuscript.
